# Differential transcript expression between the microfilariae of the filarial nematodes, *Brugia malayi *and *B. pahangi*

**DOI:** 10.1186/1471-2164-11-225

**Published:** 2010-04-07

**Authors:** Michael M Kariuki, Leonard B Hearne, Brenda T Beerntsen

**Affiliations:** 1 Department of Veterinary Pathobiology, College of Veterinary Medicine, University of Missouri, Columbia, MO 65211, USA; 2 Department of Statistics, College of Arts and Science, University of Missouri, Columbia, MO 65211, USA

## Abstract

**Background:**

*Brugia malayi *and *B. pahangi *are two closely related nematodes that cause filariasis in humans and animals. However, *B. pahangi *microfilariae are able to develop in and be transmitted by the mosquito, *Armigeres subalbatus*, whereas most *B. malayi *are rapidly melanized and destroyed within the mosquito hemocoel. A cross-species microarray analysis employing the *B. malayi *V2 array was carried out to determine the transcriptional differences between *B. malayi *and *B. pahangi *microfilariae with similar age distribution.

**Results:**

Following microarray data analysis, a list of preferentially expressed genes in both microfilariae species was generated with a false discovery rate estimate of 5% and a signal intensity ratio of 2 or higher in either species. A total of 308 probes were preferentially expressed in both species with 149 probes, representing 123 genes, in *B. pahangi *microfilariae and 159 probes, representing 107 genes, in *B. malayi *microfilariae. In *B. pahangi*, there were 76 (62%) up-regulated transcripts that coded for known proteins that mapped into the KEGG pathway compared to 61 (57%) transcripts in *B. malayi *microfilariae. The remaining 47 (38%) transcripts in *B. pahangi *and 46 (43%) transcripts in *B. malayi *microfilariae were comprised almost entirely of hypothetical genes of unknown function. Twenty-seven of the transcripts in *B. pahangi *microfilariae coded for proteins that associate with the secretory pathway compared to thirty-nine in *B. malayi *microfilariae. The data obtained from real-time PCR analysis of ten genes selected from the microarray list of preferentially expressed genes showed good concordance with the microarray data, indicating that the microarray data were reproducible.

**Conclusion:**

In this study, we identified gene transcripts that were preferentially expressed in the microfilariae of *B. pahangi *and *B. malayi*, some of which coded for known immunomodulatory proteins. These comparative transcriptome data will be of interest to researchers keen on understanding the inherent differences, at the molecular level, between *B. malayi *and *B. pahangi *microfilariae especially because these microfilariae are capable of surviving in the same vertebrate host but elicit different immune response outcomes in the mosquito, *Ar. subalbatus*.

## Background

Lymphatic filariasis is caused by the thread-like parasitic nematodes *Wuchereria bancrofti, Brugia malayi and B. timori *which are transmitted by mosquitoes. *B. malayi *and *B. timori *are the main cause of Brugian lymphatic filariasis in humans and *B. pahangi *causes filariasis in domestic cats, dogs and wild animals. The periodic form of *B. malayi *primarily infects humans whereas the sub-periodic form is zoonotic [1]. Both *B. malayi *and *B. pahangi *have similar life cycles in their natural vertebrate and mosquito hosts. The adult worms of both species reside in the lymphatic vessels of infected vertebrates and, when mature, produce sheathed microfilariae that enter the blood stream. When a competent mosquito acquires microfilariae in a blood meal, the microfilariae develop within the mosquito into third stage larval forms that are infective to vertebrates.

The *B. malayi *and *B. pahangi *microfilariae are morphologically similar, but genetically these worms can be differentiated by restriction site polymorphism within specific regions of repeated DNA sequences [2-4]. Physiologically, both *B. malayi *and *B. pahangi *microfilariae are able to develop to infective stage larvae in *Anopheles quadrimaculatus *and *Aedes aegypti *[5]. However, only *B. pahangi *microfilariae are able to develop in and be transmitted to the vertebrate host by the natural vector, *Armigeres subalbatus*, whereas most of the *B. malayi *microfilariae are rapidly and effectively destroyed in the hemocoel by melanotic encapsulation [6-8]. Melanin synthesis in mosquitoes involves a series of complex biochemical reactions that are triggered in response to either injury to the insect body or by molecules on the surface of invading microbial pathogens [9].

The antigens on the surface of *B. malayi *and *B. pahangi *microfilariae have been found to be similar, with antibodies raised against surface antigens of one species cross-reacting with surface antigens from the other species [10]. However, the protein profile of microfilaria surface antigens seen on SDS-PAGE analysis shows differences in molecular weight of some surface antigens, indicating that there are species-specific molecules on the *B. malayi *and *B. pahangi *microfilariae surfaces [10]. The two outer layers of the microfilariae, the sheath and cuticle, play an important role in host-parasite interactions and have been shown to contain carbohydrate epitopes on their surfaces [11,12]. Immunochemical studies have shown that during *B. malayi *microfilariae maturation, the composition of the surface antigens changes and results in differences in mosquito infectivity between immature and mature microfilariae [13]. With regard to mosquito immunity, carbohydrate molecules and other pathogen-associated molecules located on the surface on invading pathogens are recognized by mosquito pattern recognition receptors in a species-specific manner and lead to the activation of mosquito defenses such as phagocytosis and melanotic encapsulation [14]. It is therefore possible that subtle differences in the carbohydrate or protein content of the *B. malayi *and *B. pahangi *microfilariae surface may be responsible for the species-specific melanotic encapsulation response of *Ar. subalbatus *towards *B. malayi *and *B. pahangi *microfilariae. It is also a possibility that *B. pahangi *microfilariae secrete molecules that modulate the melanotic encapsulation response mounted by *Ar. subalbatus*.

During the past decade, microarray analysis has paved the way for thorough studies of gene expression [15,16]. More recently, cross-species hybridization analysis has been used as a way to investigate differences between two closely related species [17-19]. In this study, we sought to determine the differences between *B. pahangi *and sub-periodic *B. malayi *microfilariae at the transcriptional level using cross-species microarray analysis. First, we identified the global molecular differences that exist between the transcriptomes of the microfilariae of these two species. Second, we identified transcripts that are preferentially expressed in one species and whose products are likely to be secreted or located on the surface of the microfilariae. Proteins that are secreted by microfilariae or located on their surface are likely to play a significant role in the interaction of microfilariae with their vertebrate and mosquito hosts.

## Results

### Sequence comparison between *B. pahangi *and *B. malayi *genes

**Figure 1:**
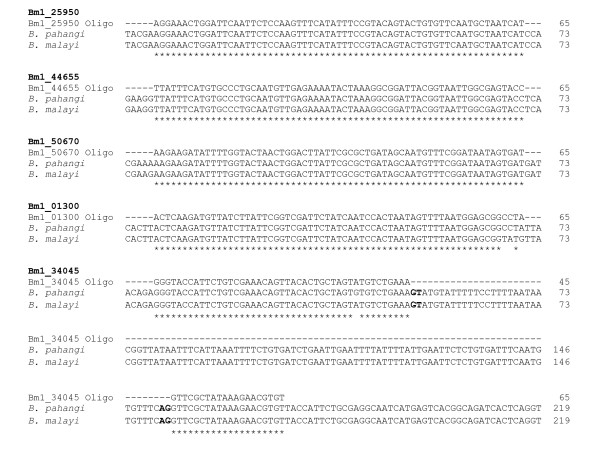
**Sequence alignment of five probes against the PCR products of *B. pahangi *and *B. malayi***. Primers designed to flank the probe sequence of five probes on the BmV2 chip that had three to ten times higher signal intensities in *B. malayi *microfilariae than in *B. pahangi *were used for PCRs with *B. pahangi *and *B. malayi *genomic DNA as templates and the PCR product was sequenced. The asterisks indicate identical nucleotides in the aligned sequences. For Bm1_34045, the consensus dinucleotides at either ends of the intron, GT at the 5' end and AG at the 3' end, are shown in bold for both the *B. pahangi *and *B. malayi *genes.

We selected twenty-two nucleotide sequences of *B. pahangi *genes, complete or partial, deposited in GenBank and compared their nucleotide sequences to their counterpart genes in *B. malayi *(Table 1). The alignment results showed that, on average, *B. malayi *and *B. pahangi *genes had 97% nucleotide sequence identity. During cross-species microarray hybridizations, a sequence mismatch between the probe and the target sequence in one species can lead to inaccurate interpretations of target levels between the species. In this study, we sought to determine whether there were sequence differences between *B. malayi *and *B. pahangi *genes particularly in the sequence used as probes and spotted on the array. To this end, we randomly selected five *B. malayi *genes whose probes were present on the BmV2 array and designed primers that flanked the probe sequence. We carried out PCRs using these primers on *B. malayi *and *B. pahangi *genomic DNA templates and sequenced the PCR products. The alignment results showed that the oligonucleotide sequence of the selected probes were a match for both the *B. malayi *and *B. pahangi *genes (Figure 1). In one case, the bm.02018 probe derived from Pub Locus Bm1_34045 appears to straddle an exon-intron junction as evidenced by the consensus dinucleotides found at each end of the intron, GT at the 5' end and AG at the 3' end of the intron [20].

**Table 1:** Nucleotide sequence similarity between *B. pahangi *and *B. malayi *genes.

Accession #	*Brugia *Gene	Identical	Total	% Identity	Gaps
AF013991	TGF-b type I receptor	1912	1942	98	2
U45314	Microfilarial sheath protein	1883	2007	93	55
AJ005784	Heat shock protein 90	1796	1822	98	6
AAS18673	Gamma-glutamyl transpeptidase precusor	1756	1773	99	0
X69128	Glutathione peroxidase	1504	1582	98	45
M27191	Heat shock protein 70 (hsp70) mRNA	1202	1228	97	7
AF031819	Cathepsin L-like cysteine protease	1133	1188	95	10
U59690	Chitinase partial CDs	1126	1153	97	8
AJ249374	*Brugia pahangi *col-1 gene cuticular collagen	864	870	99	0
AF091046	Nuclear hormone receptor DNA binding protein	772	780	98	3
AJ130821	Alt-1 protein	617	636	97	3
AJ271611	Cytochrome oxidase subunit I partial CDs	573	632	90	6
X76283	Extracellular Cu/Zn-superoxide dismutase	550	584	94	17
X87901	Small heat shock protein	525	534	98	0
X76284	Cytosolic Cu/Zn-superoxide dismutase	483	493	97	2
AY050257	Fatty acid retinoid binding protein precursor (far-1) mRNA	474	477	99	0
X91065	Cytidine deaminase	399	402	99	0
EF413918	Myosin partial CDs	384	390	98	1
X95663	Elongation factor 1 alpha partial CDs	365	375	97	1
EF413922	Pyruvate dehydrogenase partial CDs	338	339	99	0
AJ012618	*Brugia pahangi *cut-1 gene	244	252	96	0
AJ224966	*Brugia pahangi *mRNA for tropomyosin	170	171	99	0

	Total	19070	19630	(Avg) 97	

The partial or complete nucleotide sequences of twenty-two *B. pahangi *genes deposited in GenBank were run on the NCBI's nucleotide BLAST program. The output of the BLAST analysis assigned to the corresponding *B. malayi *gene is shown above.

### Transcript expression profiles of *B. pahangi *and *B. malayi *microfilariae genes

A draft of the whole *B. malayi *genome was recently published [21] and of the 15412 *B. malayi *probes on the BmV2 array, 11975 probes had matching sequences. Of these 11975 probes, statistical analysis of the microarray data revealed that a total 877 probes (7%) had signal intensities above threshold in at least one species with 504 probes higher in *B. pahangi *and 373 probes higher in *B. malayi*. Probes with FDR estimates of 5% and signal intensity ratios of 2 or higher, with respect to either species, were considered preferentially expressed. There were 149 probes that had signal intensity ratios of 2 or higher in *B. pahangi *microfilariae and 159 probes in *B. malayi *microfilariae. The 149 probes identified in *B. pahangi *represented 123 genes and the 159 probes identified in *B. malayi *represented 107 genes (Table 2) as some genes were represented by more than one probe on the BmV2 array.

**Table 2:** Overall number of preferentially expressed transcripts in *B. pahangi *and *B. malayi *microfilariae.

Microarray data treatment at 5% FDR estimate	Bp/Bm	Bm/Bp	Total
Probe signal intensity ratio >=1	504 (57%)	373 (43%)	877
Probe signal intensity ratio >=2	149 (48%)	159 (52%)	308
^a ^Genes with signal intensity ratio >=2	123 (53%)	107 (47%)	230

^a ^Some genes were represented by more than one probe on the BmV2 array. The significance cut-off was set at a 5% false discovery rate (FDR) estimate. Bp, *B. pahangi*; Bm, *B. malayi*.

### Functional classification of preferentially expressed gene transcripts

Not all the genes represented on the BmV2 array had Gene Ontology (GO) assignments. Of the 123 gene transcripts found to be preferentially up-regulated in *B. pahangi *microfilariae, 76 (62%) of them coded for known proteins that mapped into KEGG pathways and the remaining 47 (38%) transcripts encoded mostly hypothetical proteins of unknown function. Of the 76 known genes, there were 14 genes involved in cellular processes, 9 genes in environmental information processes, 37 genes in genetic information processing and 16 genes in metabolism (Additional File 1).

**Figure 2:**
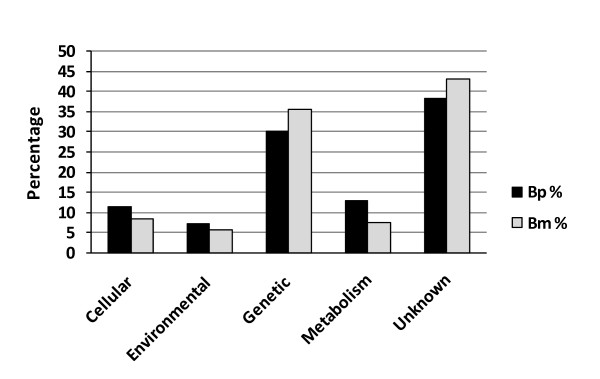
**KEGG pathway classification of preferentially expressed genes in *B. pahangi *and *B. malayi *microfilariae**. The genes that were classified as up-regulated in either species were manually mapped into the KEGG pathway groups. Cellular; Cellular Processes, Environmental; Environmental Information Processing, Genetic; Genetic Information Processing.

Of the 107 gene transcripts preferentially up-regulated in *B. malayi *microfilariae, 61 (57%) of them coded for known proteins that mapped into KEGG pathways and the remaining 46 (43%) transcripts encoded mostly hypothetical proteins of unknown function. Of the 61 known genes, there were 9 genes involved in cellular processes, 6 genes in environmental information processes, 38 genes in genetic information processing and 8 genes in metabolism (Additional File 2). A comparison of the preferential expressed gene transcripts in both *B. pahangi *and *B. malayi *based upon their overall KEGG pathway association is shown in Figure 2.

### Predicted localization of proteins encoded by preferentially expressed gene transcripts

The proteins coded for by the transcripts found to be preferentially up-regulated in *B. malayi *and *B. pahangi *were grouped according to whether they were secretory or non-secretory as indicated by the SignalP [22] predictions provided on the BmV2 array annotation. Of the 123 genes up-regulated in *B. pahangi *microfilariae, 27 genes (22%) coded for proteins containing a signal sequence on the N-terminus implying that these proteins associate with the secretory pathway while the remaining 96 genes (78%) coded for proteins without a signal sequence. Of the 27 proteins associated with the secretory pathway, TargetP [23] predictions indicate that 3 proteins are likely to be located in the mitochondria, 16 proteins are likely to be secreted and 8 have an unknown destination (Table 3).

**Table 3:** Localization predictions for proteins encoded by preferentially expressed transcripts in *B. pahangi *microfilariae.

Pub Locus	Annotation	SignalP	TargetP^a^	PSORTII	ProtComp
Bm1_49810	Conserved hypothetical protein	Signal	mitochondria (5)	CY	PM
Bm1_44755	RE21922p, putative	Signal	mitochondria (5)	ER	PM
Bm1_23875	Hypothetical protein	Signal	mitochondria (5)	GO	ER
Bm1_03995	Serpin (BmSERPIN), putative	Signal	secreted (1)	ER	ER
Bm1_02070	Serpin, putative	Signal	secreted (1)	ER	ER
Bm1_32890	Histidine acid phosphatase family protein	Signal	secreted (1)	ER	MT
Bm1_07905	Metallophosphoesterase 1, putative	Signal	secreted (1)	ER	PM
Bm1_42865	*Brugia malayi *antigen, putative	Signal	secreted (1)	EX	EX
Bm1_40635	Conserved hypothetical protein	Signal	secreted (1)	EX	EX
Bm1_37310	FKBP-type peptidyl-prolyl cis-trans isomerase-13, BmFKBP-13	Signal	secreted (1)	EX	EX
Bm1_47735	Lipase family protein	Signal	secreted (1)	EX	EX
Bm1_49635	Protein C17H12.11, putative	Signal	secreted (1)	EX	EX
Bm1_53990	Zinc finger, C2H2 type family protein	Signal	secreted (1)	EX	NU
Bm1_39915	Transthyretin-like family protein	Signal	secreted (2)	CY	EX
Bm1_00735	Hypothetical protein	Signal	secreted (2)	CY	NU
Bm1_50605	Ribonuclease T2 family protein	Signal	secreted (2)	ER	EX
Bm1_28420	DnaJ protein, putative	Signal	secreted (2)	EX	EX
Bm1_56195	Transforming growth factor b homolog, putative	Signal	secreted (2)	EX	EX
Bm1_33495	Probable protein disulfide isomerase A6 precursor, putative	Signal	secreted (3)	EX	ER
Bm1_25940	Serpin, putative	Signal	unknown (2)	ER	EX
Bm1_35905	Hypothetical protein	Signal	unknown (3)	ER	PM
Bm1_43565	MBOAT family protein	Signal	unknown (3)	PM	ER
Bm1_18845	GRIM-19 protein	Signal	unknown (4)	CY	GO
Bm1_37335	Ankyrin-related unc-44, putative	Signal	unknown (4)	NU	EX
Bm1_40630	Hypothetical protein	Signal	unknown (5)	CY	PM
Bm1_24145	ABC1 family protein	Signal	unknown (5)	ER	PE
Bm1_29610	Hypothetical protein	Signal	unknown (5)	EX	NU

^a ^Values in parentheses from TargetP results represent the reliability class (RC), from 1 to 5, where 1 indicates the strongest prediction. For PSORTII, location with the highest percentage score is shown. For ProtComp, the integral prediction of protein location is shown. CY, cytoplasm; ER, endoplasmic reticulum; EX, extracellular; GO, Golgi apparatus; NU, nuclear; PE, peroxisome; PM, plasma membrane; MT, mitochondria.

Similarly, in *B. malayi*, SignalP predictions on the BmV2 array annotation suggested that, of the 107 genes up-regulated in *B. malayi *microfilariae, 39 genes (36%) coded for proteins that associate with the secretory pathway and the remaining 68 genes (64%) were denoted as non-secretory because of the lack of a signal sequence on the N-terminus of the protein. Of the 39 proteins associated with the secretory pathway, TargetP predictions suggested that 2 proteins were likely to be localized in the mitochondria, 32 proteins were likely to be secreted and 5 had an unknown destination (Table 4).

**Table 4:** Localization predictions of proteins encoded by preferentially expressed transcripts in *B. malayi *microfilariae.

Pub Locus	Annotation	SignalP	TargetP^a^	PSORTII	ProtComp
Bm1_12070	K+ channel tetramerisation domain containing protein	Signal	mitochondria (5)	GO	PM
Bm1_02255	Zinc finger, C2H2 type family protein	Signal	mitochondria (5)	NU	NU
Bm1_14030	Hypothetical protein	Signal	secreted (1)	CY	EX
Bm1_56305	Leucyl aminopeptidase, putative	Signal	secreted (1)	ER	EX
Bm1_50670	Potassium channel chain n2P18 homolog, putative	Signal	secreted (1)	ER	EX
Bm1_30525	Hypothetical protein	Signal	secreted (1)	EX	EX
Bm1_33955	Hypothetical protein	Signal	secreted (1)	EX	EX
Bm1_19100	Major microfilarial sheath protein precursor, putative	Signal	secreted (1)	EX	EX
Bm1_10870	Zinc finger, C2H2 type family protein	Signal	secreted (1)	NU	CY
Bm1_09090	Zinc finger, C2H2 type family protein	Signal	secreted (1)	NU	ER
Bm1_55930	Nematode cuticle collagen N-terminal domain containing protein	Signal	secreted (1)	NU	EX
Bm1_18365	Zinc finger, C2H2 type family protein	Signal	secreted (1)	NU	EX
Bm1_22980	Zinc finger, C2H2 type family protein	Signal	secreted (1)	NU	EX
Bm1_35265	Zinc finger, C2H2 type family protein	Signal	secreted (1)	NU	EX
Bm1_34385	Hypothetical protein	Signal	secreted (1)	NU	GO
Bm1_04225	Zinc finger, C2H2 type family protein	Signal	secreted (1)	NU	GO
Bm1_11185	Zinc finger, C2H2 type family protein	Signal	secreted (1)	NU	GO
Bm1_13985	Zinc finger, C2H2 type family protein	Signal	secreted (1)	NU	GO
Bm1_47425	Zinc finger, C2H2 type family protein	Signal	secreted (1)	NU	GO
Bm1_21005	Hypothetical protein	Signal	secreted (1)	NU	MT
Bm1_22820	Hypothetical protein	Signal	secreted (2)	CY	EX
Bm1_45345	Hypothetical 31.4 kDa protein T19C3.2 chromosome III, putative	Signal	secreted (2)	ER	EX
Bm1_22110	Hypothetical protein	Signal	secreted (2)	ER	PM
Bm1_33830	Prismalin-14, putative	Signal	secreted (2)	MT	EX
Bm1_23300	Zinc finger, C2H2 type family protein	Signal	secreted (2)	NU	EX
Bm1_03355	Hypothetical protein	Signal	secreted (3)	CY	EX
Bm1_08145	Zinc finger, C2H2 type family protein	Signal	secreted (3)	NU	NU
Bm1_44495	Hypothetical protein	Signal	secreted (4)	CY	EX
Bm1_00295	Hypothetical protein	Signal	secreted (4)	ER	PM
Bm1_47450	Conserved hypothetical protein, putative	Signal	secreted (4)	GO	EX
Bm1_31055	RNA recognition motif containing protein, putative	Signal	secreted (4)	NU	EX
Bm1_15340	Zinc finger, C2H2 type family protein	Signal	secreted (4)	NU	NU
Bm1_03710	Zinc finger, C2H2 type family protein	Signal	secreted (5)	NU	EX
Bm1_05155	Zinc finger, C2H2 type family protein	Signal	secreted (5)	NU	EX
Bm1_01300	Alpha amylase, catalytic domain containing protein	Signal	unknown (1)	CY	EX
Bm1_25950	Hypothetical protein	Signal	unknown (2)	ER	EX
Bm1_44655	Fukutin, putative	Signal	unknown (2)	ER	PM
Bm1_01585	Fatty acid elongation protein 3, putative	Signal	unknown (5)	ER	EX
Bm1_56360	Nematode cuticle collagen N-terminal domain containing protein	Signal	unknown (5)	NU	EX

^a ^Values in parentheses from TargetP results represent the reliability class (RC), from 1 to 5, where 1 indicates the strongest prediction. For PSORTII, location with the highest percentage score is shown. For ProtComp, the integral prediction of protein location is shown. CY, cytoplasm; ER, endoplasmic reticulum; EX, extracellular; GO, Golgi apparatus; NU, nuclear; PE, peroxisome; PM, plasma membrane; MT, mitochondria.

The 27 *B. pahangi *and 39 *B. malayi *predicted secretory proteins coded for by preferentially expressed transcripts were further analyzed by PSORTII [24] and ProtComp http://www.softberry.com; two protein algorithm that give more detailed protein localization predictions. Of the 27 *B. pahangi *proteins, 20 proteins were predicted to be either extracellular or on the plasma membrane by PSORTII or ProtComp with the remaining 7 proteins being predicted to be in the cytoplasm, Golgi apparatus, endoplasmic reticulum, peroxisome, mitochondria or nucleus (Table 3). Of the 39 *B. malayi *proteins with signal sequences, 28 proteins were predicted to be either extracellular or on the plasma membrane by PSORTII or ProtComp with the remaining 11 proteins suggested as being localized in the cytoplasm, Golgi apparatus, endoplasmic reticulum, peroxisome, mitochondria or nucleus (Table 4).

### Validation of microarray data by real-time PCR analysis

**Figure 3:**
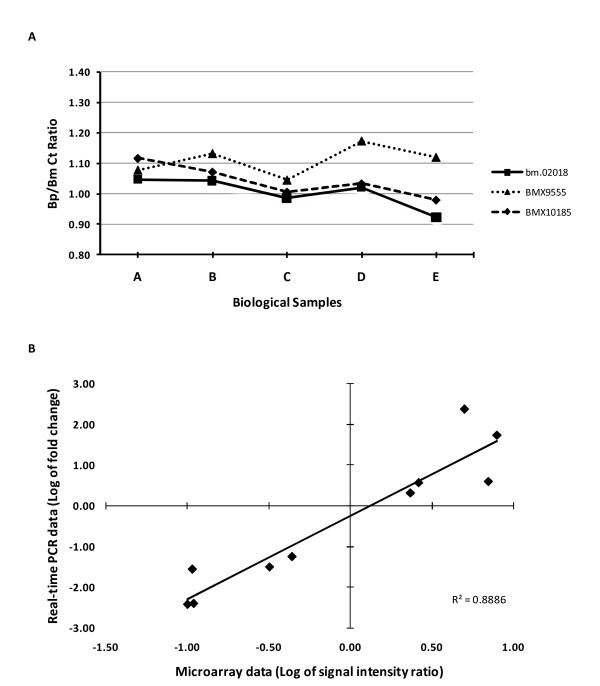
**Comparison of real-time PCR and microarray data of ten randomly selected genes**. Real-time PCR analysis was performed on cDNA derived from five biological samples of *B. pahangi *and *B. malayi *microfilariae with similar age distribution. (A) The ratio of Ct values obtained following real-time PCR analysis using primers flanking three probes shown to have similar signal intensities in the microarray data. Probe bm.02018, which represented Pub locus Bm1_34045, was assigned as the endogenous control gene. (B) Comparison of log-transformed microarray data (three biological samples) and real-time PCR data (five biological samples) of the ten representative genes showing good agreement of expression values obtained from both methodologies. Biological samples used for real-time PCR analysis were independent of those used for microarray analysis.

To verify the microarray data, 13 *Brugia *genes were selected for real-time PCR analysis (Table 5). The genes were selected based on the signal intensities of their probes, in either species, from the microarray data. Five of the selected genes had higher signal intensities in *B. pahangi*, five in *B. malayi *and the remaining three had similar signal intensities in both species. First, it was important to identify a gene with equal transcript levels in both species that was to be use as an endogenous reference. To this end, primers were designed that amplified between 200-300 base pair fragments of the Pub Locus represented by the three probes, bm.02018, BMX9555 and BMX10185, that showed similar signal intensities in the microarray data. Real-time PCR analysis was performed using these primers on cDNA derived from five biological samples of *B. pahangi *and *B. malayi *microfilariae with similar age distribution. Of the three probes with similar signal intensities in both species on the microarray data, the bm.02018 probe, which was derived from the Pub Locus Bm1_34045, was found to have a Ct ratio closest to the value one, across all five biological samples (Figure 3A). For this reason, Bm1_34045 was assigned as the endogenous reference to which the remaining ten genes were normalized. The comparative Ct method was used to determine preferentially expressed transcripts [25]. The *Brugia *microfilaria samples used for real-time PCR were independent of those used in the microarray analysis. The comparison of real-time PCR data to that obtained in the microarray analysis showed the same trend and a strong correlation between the data obtained by the two methods using different biological samples (Figure 3B), suggesting that the transcriptional profiles derived from the microarray data are accurate and reproducible.

**Table 5:** Sequence of gene-specific primers used for real-time PCR analysis.

Pub locus	Forward primer	Reverse primer
Bm1_49585	GACCGAAACAAGAAAGGAAGCCGA	ACTTTCATCATCATTATGTCGTCGCG
Bm1_11050	GATGAAACGTTTCATGAGATG	GATAATCCTCCAAAACTACCG
Bm1_02070	AATAATTGGATTTCGAGTGAGAC	TTCATGCATAATTACCAGTTTCG
Bm1_28420	AAGGCTTCGATATGTTTCCATC	CTAACCACTAGTACATATTGTCT
Bm1_42865	AAACATGCAGTTAATGCACGAG	TGCTTGCAATTCATTCCTCACT
Bm1_19100	GCCACAAGGTATGCAACCACA	GTGTCTGCGTGGGTGGTAAC
Bm1_25950^a^	GCTAACTTCAATGCACTTAGTG	AAATAAGAAACAACGTTTGATACAG
Bm1_01300^a^	CAATTTTCAGACTCAATCTACTG	AGGTAAGTATTTGTCAACGTTTG
Bm1_44655^a^	GTACGAAAGCAAGGATTCTGCT	CAGAATACTTTGTAGGTATCATTC
Bm1_50670^a^	GTATTAGCTCAAGCAATGGAAG	CTAACTACTATCATCACTATTATC
bm.02018^ab^	AGAATCAAATGTCCAGGAAG	CTGTGAGAGGAAGATAGTC
BMX9555^b^	TTGATGTAGATGCTTTAACAAGTGCTGCTC	AAGAAATTGTCGACAAAGTCCGCAAG
BMX10185^b^	CCGCACAACAATCCTCACTTGCT	TGTGTTACTGCTATTAACATCCTCACTGCC

Each primer is written in the 5' to 3' direction.

^a ^Indicates the genes whose probe regions were amplified and sequenced using the primers shown.

^b ^Indicates the probes whose primers were used to determine a suitable endogenous control for real-time PCR analysis.

## Discussion

This study was a cross-species comparison of gene transcription between the microfilariae of *B. malayi *and *B. pahangi*. The *B. pahangi *and *B. malayi *microfilariae used in this study were both obtained from similar micro-environments; the intra-peritoneal cavity of experimentally-infected jirds. Therefore, it would be safe to assume that the difference in the transcriptional profile of each nematode species was intrinsic to each species and not due to micro-environmental influence.

In order to absorb any possible variation in microfilaria gene transcription between the two species due to the host immune responses or slight differences in microfilaria age distribution, independent biological samples of *B. malayi *and *B. pahangi *microfilariae were collected from eight pairs of jirds and used in this study. Three paired biological samples were used for the microarray analysis and five paired biological samples, different from those used for microarray analysis, were used in the real time PCR analysis to confirm the validity of the microarray data. For the microarray analysis, there were 3 technical replicates for each of the 3 biological samples, resulting in the use of nine microarray slides. Only those genes with similar signal intensity values across all nine microarrays, and by extension all 3 biological samples, were further analyzed and are shown on the gene lists. As a result, any influence on microfilaria gene transcription arising from subtle differences in microfilaria age distribution between the two species, immune pressure on the microfilaria by the vertebrate immune response, or artifacts of sample preparation was therefore greatly reduced.

The BmV2 array used in this study consisted of probes designed from *B. malayi *genomic gene models and ESTs. Since the probes were an exact match for *B. malayi *genes and not necessarily proven to be so for the corresponding *B. pahangi *genes, one could assume that hybridizations on the BmV2 array would be biased towards *B. malayi*. Sequence divergence in some genes, particularly a sequence mismatch within the oligonucleotides used as a probe, could influence the hybridization kinetics and result in misleading data with respect to a cross-species differential gene transcription comparison [26,27].

In this study, we found that the *B. pahangi *genes with sequences deposited in GenBank had a high sequence identity to their counterpart *B. malayi *genes. In addition, with respect to this study, we intentionally selected five probes on the BmV2 chip that had a *B. malayi *to *B. pahangi *signal intensity ratio between three to ten and were able to show that the sequence of these oligonucleotide probes matched the cognate regions on both the *B. malayi *and *B. pahangi *genes. In addition, the microarray data as a whole did not show any bias towards *B. malayi *in the total number of preferentially up-regulated gene transcripts. Indeed, there were slightly more gene transcripts that were up-regulated in *B. pahangi *than in *B. malayi *microfilariae. These findings taken together suggest that *B. pahangi *and *B. malayi *may have almost identical gene sequences to the extent that it would be possible to obtain biologically meaningful gene transcription profiles of *B. malayi *and *B. pahangi *microfilariae using the BmV2 array with minimal bias toward *B. malayi*.

That said, it is possible that there are *B. pahangi *genes whose nucleotide sequence in the coding region differs slightly from that of their orthologs in *B. malayi*, hence introducing probe-bias towards *B. malayi *transcripts in a cross-species hybridization microarray analysis. For this reason, this study focused mainly on genes whose transcripts appeared to be preferentially up-regulated in *B. pahangi *microfilariae; a conservative approach bearing in mind that genes unique to *B. pahangi *and those with extensive mismatch between the probe and target cDNA sequence may not have been detected in the microarray analysis.

The genes that were classified as up-regulated in either species were manually mapped into the KEGG pathway groups. The composition of genes in some of the KEGG pathway groupings of up-regulated genes in *B. pahangi *and *B. malayi *microfilariae revealed some noteworthy differences. For instance in *B. malayi *microfilariae, transcripts coding for proteins containing a zinc finger domain accounted for 24 of the 34 (70%) transcripts that code for proteins involved in the processing of genetic information compared to only 2 out of 24 (8%) in *B. pahangi *microfilariae. The zinc finger domain-containing proteins are thought to be mainly involved with the regulation of transcription [28]. The reason for the bias in *B. malayi *towards having higher numbers of up-regulated transcripts of zinc finger domain-containing proteins is at present not clear.

Melanotic encapsulation is a major antimicrobial innate immune defense mechanism in insects that involves the rapid deposition of melanin around invading microbes [9]. Molecules on the surface of invading pathogens are thought to be one of the triggers of melanin synthesis in insects. The proteins and molecules on the surfaces of *B. malayi *and *B. pahangi *microfilariae have been shown to be similar with only slight differences in the molecular weights of some proteins [10]. Microfilarial proteins likely to modulate the vertebrate and mosquito immune response are those that are likely to be secreted by or on the surface of the microfilariae. Our microarray data showed that both species had, between them, a total of sixty-six preferentially expressed transcripts, twenty seven in *B. pahangi *and thirty nine in *B. malayi*, that coded for proteins likely to be secreted by or on the surface of the microfilariae. As microfilariae are exposed to both vertebrate and mosquito immune responses in quick succession, it is possible that some of these proteins may have a dual function in helping to promote microfilaria survival in midst of a human or mosquito immune response. Among transcripts preferentially transcribed in *B. pahangi *microfilariae and identifiable as coding for known immunomodulatory proteins were a putative homolog of the transforming growth factor-beta (TGF-beta) [29], three putative serpins [30] and two FKBP-type peptidyl-prolyl cis-trans isomerase proteins [31,32].

Human TGF-beta is a ubiquitous protein that plays a role in most biological processes. With regard to immunity, TGF-beta acts as an inhibitory cytokine that has an important role, together with interleukin-10, in down regulating immunity and hence maintain self-tolerance and immune homeostasis [29,33]. The *B. malayi *ortholog of TGF-beta, designated as Bm-TGH-2, is expressed mainly in mature adult parasites and the microfilariae; two of the nematode's life stages that are constantly exposed to the human immune response factors [34]. It is, therefore, plausible that the Bm-TGH-2 gene in *B. pahangi *may mimic the functions of human TGF-beta by modulating the vertebrate immune response, thereby promoting parasite survival within the vertebrate host [34].

In melanoma cells, TGF-beta has been shown to significantly inhibit melanin synthesis by increasing the rate of degradation of tyrosinase, a rate-limiting enzyme in the melanogenesis pathway [35,36]. In the *Brugia *microfilariae-*Ar. subalbatus *interaction, most *B. pahangi *microfilariae are not melanized whereas the *B. malayi *microfilariae are melanized and destroyed in the mosquito hemocoel [7], suggesting that the *B. pahangi *microfilariae actively subvert or are deficient of the elements that trigger the synthesis of melanin in *Ar. subalbatus*. In light of our microarray data showing that *B. pahangi *microfilariae had significantly higher transcript levels of the TGF-beta homolog than *B. malayi *microfilariae, it is possible that *B. pahangi*-derived TGF-beta may negatively modulate the synthesis of melanin, essential in the melanization immune response in *Ar. subalbatus*, by a similar mechanism as that shown to occur in melanoma cells.

Serpins are proteins that inhibit the serine proteases that control proteolytic cascades in various cellular processes. In vertebrates, serpins function to mitigate the damage caused by proteases involved in the body's immune response to injury such as inflammation and coagulation [30,37]. In the case of insects, serpins have been shown to negatively regulate the proteolytic cascade of serine proteases that activate phenoloxidase, a key enzyme in the biochemical synthesis of melanin [38,39]. The effect, if any, that microfilaria-derived serpins have on the vertebrate immune system remains unclear [40,41]. In the case of mosquito-parasite interactions, RNAi studies have shown that mosquito-derived serpins influence the outcome of malaria parasite infections in a parasite species-specific manner whereby the knockdown of *Anopheles gambiae *serpin 2 (SRPN2) leads to markedly reduced numbers of *P. berghei oocysts* in the *A. gambiae *midgut [42] but has no effect on the development of *P. falciparum *oocysts [43]. To date, there have been no reported functional studies on the effect of *Brugia *microfilaria serpins on the mosquito melanotic immune response. According to the recently published draft of the *B. malayi *genome [44], there are thirteen serpin locus tags implying that there may be up to thirteen possible serpin genes in *B. malayi*, however only 8 were represented on the BmV2 array. The microarray results in this study showed that *B. pahangi *microfilariae had two to five times higher transcript levels of three putative serpins than did *B. malayi *microfilariae. It may be that these *B. pahangi*-derived serpins modulate the melanization immune response in *Ar. subalbatus*.

## Conclusion

In nature, the microfilariae of both species encounter the vertebrate and insect immune responses in fairly quick succession following their ingestion in a blood meal by a mosquito. In order for the microfilariae to survive in both the vertebrate and mosquito hosts, it is conceivable that the microfilariae possess a mechanism that functions to modulate both the vertebrate and mosquito immune systems. In the current study, we identified transcripts that are preferentially expressed in *B. pahangi *and *B. malayi *microfilariae. These comparative transcriptome data will be of interest to researchers keen on understanding the intrinsic difference, at the molecular level, between *B. malayi *and *B. pahangi *microfilariae especially because these microfilariae elicit such different immune response outcomes in the mosquito, *Ar. subalbatus*. In addition, we further identified transcripts that encode for proteins that are predicted to be secreted or located on the surface of microfilariae and which could have a significant and dual role in the successive interaction of the microfilaria with the vertebrate and mosquito hosts. Consequently, some of these proteins may serve as ideal targets for intervention strategies against the transmission of Brugian lymphatic filariasis.

## Methods

### Parasite material

*B. pahangi *and sub-periodic *B. malayi *microfilariae of similar age distribution, harvested from the intra-peritoneal cavity of experimentally infected jirds, were provided by the NIH/NIAID Filariasis Research Reagent Repository Center (FR3), Parasite Resources Division, College of Veterinary Medicine, University of Georgia, Athens, GA. The FR3 protocol for the experimental infection of jirds with *B. malayi *and *B. pahangi *and the subsequent collection of microfilariae was as follows. Approximately 1000 each of infective third-stage larvae (L3) of *B. malayi *and *B. pahangi*, collected from infected *Ae. aegypti*, were injected on the same day or not more than a week apart into the peritoneal cavity of recipient jirds. The microfilariae were recovered from the peritoneal cavity of recipient jirds by peritoneal washing using RPMI 1640 medium. On each occasion, the *B. malayi *microfilariae were recovered from the peritoneal cavity of a single euthanized jird as were *B. pahangi *microfilariae. The microfilariae recovered from the peritoneal cavity of infected jirds were transferred to a 50 ml centrifuge tube and washed five times with Hanks' Buffered Salt Solution (HBSS). An aliquot of the microfilariae was removed and examined using a microscope to ascertain microfilaria viability and number.

### Microarray slide

The *B. malayi *Version 2 oligonucleotide array (BmV2) was obtained from the NIH/NIAID FR3, Molecular Resources Division, Clark Science Center, Smith College, Northampton, MA. The BmV2 array was comprised of 18104 probes (65 mer) derived from The Institute for Genomic Research (TIGR) databases. There were 15412 probes derived from the extensive TIGR *B. malayi gene *indices, genomic gene models and EST database, 1016 probes derived from the TIGR *Onchocerca volvulus *gene indices, 872 probes from the TIGR *W. bancrofti *gene indices and EST clusters, and 804 probes from the genome of the *Wolbachia *endosymbiont.

### RNA preparation for microarray analysis

Total RNA was isolated from three independent biological samples of *B. malayi *and *B. pahangi *microfilariae using TRIzol reagent (Invitrogen, CA) as per the manufacturer's instructions with minor modifications to the homogenization step. Briefly, approximately two to three million microfilariae of each species were re-suspended in TRIzol reagent, a 3.0 mm stainless-steel bead (Retsch Inc., PA) was added, and the suspension was vortexed at maximum speed for 30 min at 4°C. The steel ball was removed and the suspension homogenized four times for 30 s each using a rotor-stator (PowerGen 125; Fisher Scientific, PA) fitted with a soft tissue probe (OmniTips, GA) and set at two thirds to three quarters speed. A final homogenization step was at three quarters speed for 1 min. The suspension was centrifuged at 12000 g for 10 min at 4°C. Total RNA was extracted from the supernatant following the TRIzol protocol and the concentration and purity was estimated spectrophotometrically. The total RNA quality was determined using an Agilent 2100 bioanalyzer (Agilent Technologies, CA).

### First-strand synthesis and microarray hybridization

The first-strand synthesis and microarray hybridizations were carried out at the Washington University Genome Sequencing Center, St Louis, MO. Equal amounts of cDNA prepared from total RNA isolated from three-paired *B. malayi *and *B. pahangi *microfilariae samples were competitively hybridized under stringent conditions on the BmV2 array. For RNA expression level comparison, *B. malayi *and *B. pahangi *fluorophore-specific cDNA samples were paired. First strand cDNA was generated by oligo-dT primed reverse transcription (Superscript II; Invitrogen) utilizing the 3DNA Array 900 kit (Genisphere, PA). Briefly, the fluorophore specific oligo-dT primer was added to 2 μg of total RNA and the solution incubated at 80°C for 5 min then cooled on ice for 2 min. cDNA was synthesized according to standard protocols. Each biological sample pair was re-suspended in a formamide-based hybridization buffer and Array 50dT blocker (Genisphere, PA). Two hybridizations were carried out in a sequential manner. The primary hybridization was performed by adding the cDNA samples of both microfilariae species to the microarray slide under a supported glass coverslip (Erie Scientific, NH) at 43°C for 16-20 h at high humidity. Prior to the secondary hybridization, the slide was gently submerged in 2× SSC, 0.2% SDS at 43°C for 11 min, transferred to 2× SSC at RT for 11 min followed by an incubation in 0.2× SSC at RT for 11 min and then spun dry by centrifugation. The secondary hybridization was carried out using the complimentary capture reagents provided in the 3DNA Array 900 kit (Genisphere, PA). Both the 3DNA capture reagents with Cy3 and Cy5, in a SDS-based hybridization buffer, were added to the microarray slide under a supported glass coverslip. The slide was incubated at 65°C for 4 h at high humidity in the dark. At hybridization termination, the slide was washed as described above. A dye-swap was carried out in order to alleviate concerns about gene-specific dye bias. There were three technical replicates for each of the three biological samples, resulting in a total of nine DNA microarray slides used in this study.

### Data acquisition

The slides were scanned on a Perkin Elmer ScanArray Express HT scanner (Perkin-Elmer, MA) to detect Cy3 and Cy5 fluorescence at 543 and 633 nm, respectively. Laser power was kept constant for Cy3/Cy5 scans for all slides. The gridding and analysis of images were performed using ScanArray v3.0 (Perkin-Elmer, MA).

### Statistical analysis of microarray data

Fluorescence intensities obtained for Cy3 by Cy5 for each array were converted into logarithm base 2 values and plotted. The images were assessed for plausibility, and deviations from the expected point cloud were noted. The microarray data was statistically modeled using a Linear Mixed Model to account for the array hybridization and dye variability [45,46]. The residuals from this model were then modeled for nematode species within each probe using a another Linear Mixed Model. An F-test for species difference was then computed. The p-values for differences were then sorted into ascending order and cutoff values were established for p-values using a Benjamini and Hochberg false discovery rate (FDR) of 0.05 [47,48]. The FDR is a statistically stringent manipulation that compensates for multiple hypothesis testing in data involving multiple comparisons.

### RNA preparation and real-time PCR analysis

Total RNA was isolated from five-paired *B. malayi *and *B. pahangi *microfilariae samples using the RNeasy kit (Qiagen, CA) and the contaminating genomic DNA in the total RNA was removed using the TURBO DNA-*free*™ kit (Ambion, TX). The first strand cDNA was generated using the SuperScript III First-Strand Synthesis System for RT-PCR (Invitrogen, CA). Thirteen *Brugia *genes in the microarray data were selected for validation using real-time PCR. The sequences of the selected *B. malayi *genes were retrieved from GenBank and gene-specific primers, which would amplify 200 to 400 bp fragments, were designed. The PCRs were set-up using Power SYBR Green PCR Master Mix (Applied Biosystems, CA) on 96-well plates in 20 μl reaction volumes with equal amounts of cDNA derived from *B. malayi *and *B. pahangi *microfilariae as templates. The real-time PCR was carried out on an Applied Biosystems 7300 Real-Time PCR System (Applied Biosystems, CA) and the cycle threshold values (Ct) obtained were used to determine the relative quantity of each target gene between the two *Brugia *species using the comparative Ct method [25]. Five biological samples were analyzed independently with each selected gene run in triplicate.

### Functional classification of clusters up-regulated in *Brugia *microfilariae

For this study, the probes with FDR estimates of 5% and signal intensity ratios of 2 or higher, with respect to either species, were considered preferentially expressed and prompted further analysis. In the first analysis, these preferentially expressed genes were manually grouped based how they mapped into the Kyoto Encyclopedia of Genes and Genomes (KEGG) biological pathway [49]. Additional mapping of some genes on the BmV2 array into the KEGG pathway was done based on their annotations on the BmV2 array. In the second analysis, the preferentially expressed genes that encoded proteins with a signal sequence, as predicted by the SignalP algorithm [50], were further grouped according to the suggested localization of the proteins they encode as predicted by the protein structure and localization algorithms TargetP [50] and PSORTII [24] hosted by the ExPASy proteomics server of the Swiss Institute of Bioinformatics, and the on-line protein localization algorithm, ProtComp, from Softberry Inc http://www.softberry.com/.

### Sequence comparison between *B. pahangi *and *B. malayi *genes

Twenty-two nucleotide sequences of *B. pahangi *genes, complete or partial, deposited in GenBank were selected and their nucleotide sequences compared with their orthologs in *B. malayi *using the NCBI's BLAST program. The output of the BLAST analysis was recorded. To determine whether the nucleotide sequences of the probes on the BmV2 array were identical to their cognate regions in the *B. malayi *and *B. pahangi *genes, PCRs were carried out using primers that flanked the probe sequence with *B. malayi *and *B. pahangi *genomic DNA as templates. The PCR products were purified (QIAquick PCR Purification Kit, Qiagen, CA) and sequenced from both ends using the PCR primers. The nucleotide sequences of the PCR products of both species were aligned with the probe sequence. Sequence alignment was done using the VectorNTI software (Invitrogen, CA).

## Authors' contributions

MMK prepared the RNA samples used for real-time PCR analysis and performed the real-time PCR analysis, sequence alignments, analysis of the microarray data, generation of the gene lists, and drafting of the manuscript. LBH performed the statistical analysis of the microarray data. BTB designed the study, prepared the RNA samples used in the microarray analysis, supervised all aspects of data collection and analysis, and revised the manuscript. All authors read and approved the final manuscript.

## Supplementary Material

Additional file 1 ***Brugia gene *transcripts that are up-regulated in *B. pahangi *microfilariae**. This file contains a list of genes represented by probes on the microarray that had signal intensity ratios of 2 or higher in *B. pahangi *microfilariae and a FDR estimate of 5%. These genes were considered preferentially expressed and manually mapped into the appropriate KEGG pathway. ^a ^Genes represented by more than one probe on the BmV2 array. For these genes, an average of the *p-*value and signal intensity ratio is shown. Genes in each of the KEGG pathway groups are ranked according to their ratios. Click here for file

Additional file 2 ***Brugia gene *transcripts that are up-regulated in *B. malayi *microfilariae**. This file contains a list of genes represented by probes on the microarray that had signal intensity ratios of 2 or higher in *B. malayi *microfilariae and a FDR estimate of 5%. These genes were considered preferentially expressed and manually mapped into the appropriate KEGG pathway. ^a ^Genes represented by more than one probe on the BmV2 array. For these genes, an average of the *p-*value and signal intensity ratio is shown. Genes in each of the KEGG pathway groups are ranked according to their ratios. Click here for file

## References

[B1] Mak JW Epidemiology of lymphatic filariasis Ciba Found Symp 1987 127 5 14 2885159 10.1002/9780470513446.ch2

[B2] McReynolds LA DeSimone SM Williams SA Cloning and comparison of repeated DNA sequences from the human filarial parasite *Brugia malayi *and the animal parasite *Brugia pahangi* Proc Natl Acad Sci USA 1986 83 3 797 801 10.1073/pnas.83.3.797 3003750 PMC322952

[B3] McReynolds LA Poole C Hong Y Williams SA Partono F Bradley J Recent advances in the application of molecular biology in filariasis Southeast Asian J Trop Med Public Health 1993 24 Suppl 2 55 63 7973949

[B4] Xie H Bain O Williams SA Molecular phylogenetic studies on *Brugia *filariae using Hha I repeat sequences Parasite 1994 1 3 255 260 9140492 10.1051/parasite/1994013255

[B5] Nayar JK Knight JW Vickery AC Susceptibility of *Anopheles quadrimaculatus *(Diptera: *Culicidae*) to subperiodic *Brugia malayi *and *Brugia pahangi *(Nematoda: *Filarioidea*) adapted to nude mice and jirds J Med Entomol 1990 27 3 409 411 2332882 10.1093/jmedent/27.3.409

[B6] Kobayashi MON Tsuruoka H Chigusa Y Mishima S Studies on filariasis VII: Histological observation on the encapsulated *Brugia malayi *larvae in the abdominal haemocoel of the mosquitoes, *Armigeres subalbatus* Jpn J Sanit Zool 1986 37 59 65

[B7] Beerntsen BT Luckhart S Christensen BM *Brugia malayi *and *Brugia pahangi*: inherent difference in immune activation in the mosquitoes *Armigeres subalbatus *and *Aedes aegypti* J Parasitol 1989 75 1 76 81 10.2307/3282940 2563767

[B8] Zhao X Ferdig MT Li J Christensen BM Biochemical pathway of melanotic encapsulation of *Brugia malayi *in the mosquito, *Armigeres subalbatus* Dev Comp Immunol 1995 19 3 205 215 10.1016/0145-305X(95)00005-E 8595819

[B9] Christensen BM Li J Chen CC Nappi AJ Melanization immune responses in mosquito vectors Trends Parasitol 2005 21 4 192 199 10.1016/j.pt.2005.02.007 15780842

[B10] Maizels RM Partono F Oemijati S Denham DA Ogilvie BM Cross-reactive surface antigens on three stages of *Brugia malayi, B. pahangi *and *B. timori* Parasitology 1983 87 Pt 2 249 263 10.1017/S0031182000052616 6196709

[B11] Araujo AC Souto-Padron T de Souza W Cytochemical localization of carbohydrate residues in microfilariae of *Wuchereria bancrofti *and *Brugia malayi* J Histochem Cytochem 1993 41 4 571 578 8450196 10.1177/41.4.8450196

[B12] Klonisch T Bardehle G Linder D Boschek B Schott HH Zahner H Stirm S The sheaths of *Brugia *microfilariae: isolation and composition Parasitol Res 1991 77 5 448 451 10.1007/BF00931643 1891453

[B13] Fuhrman JA Urioste SS Hamill B Spielman A Piessens WF Functional and antigenic maturation of *Brugia malayi *microfilariae Am J Trop Med Hyg 1987 36 1 70 74 2433955 10.4269/ajtmh.1987.36.70

[B14] Wang X Fuchs JF Infanger LC Rocheleau TA Hillyer JF Chen CC Christensen BM Mosquito innate immunity: involvement of beta 1,3-glucan recognition protein in melanotic encapsulation immune responses in *Armigeres subalbatus* Mol Biochem Parasitol 2005 139 1 65 73 10.1016/j.molbiopara.2004.09.009 15610820

[B15] Stoughton RB Applications of DNA microarrays in biology Annu Rev Biochem 2005 74 53 82 10.1146/annurev.biochem.74.082803.133212 15952881

[B16] DeRisi J Penland L Brown PO Bittner ML Meltzer PS Ray M Chen Y Su YA Trent JM Use of a cDNA microarray to analyse gene expression patterns in human cancer Nat Genet 1996 14 4 457 460 10.1038/ng1296-457 8944026

[B17] Bar-Or C Bar-Eyal M Gal TZ Kapulnik Y Czosnek H Koltai H Derivation of species-specific hybridization-like knowledge out of cross-species hybridization results BMC Genomics 2006 7 110 10.1186/1471-2164-7-110 16677401 PMC1482311

[B18] Nowrousian M Ringelberg C Dunlap JC Loros JJ Kuck U Cross-species microarray hybridization to identify developmentally regulated genes in the filamentous fungus *Sordaria macrospora* Mol Genet Genomics 2005 273 2 137 149 10.1007/s00438-005-1118-9 15778868

[B19] Renn SC Aubin-Horth N Hofmann HA Biologically meaningful expression profiling across species using heterologous hybridization to a cDNA microarray BMC Genomics 2004 5 1 42 10.1186/1471-2164-5-42 15238158 PMC471549

[B20] Breathnach R Benoist C O'Hare K Gannon F Chambon P Ovalbumin gene: evidence for a leader sequence in mRNA and DNA sequences at the exon-intron boundaries Proc Natl Acad Sci USA 1978 75 10 4853 4857 10.1073/pnas.75.10.4853 283395 PMC336219

[B21] Ghedin E Wang S Spiro D Caler E Zhao Q Crabtree J Allen JE Delcher AL Guiliano DB Miranda-Saavedra D Draft genome of the filarial nematode parasite Brugia malayi Science 2007 317 5845 1756 1760 10.1126/science.1145406 17885136 PMC2613796

[B22] Nielsen H Engelbrecht J Brunak S von Heijne G Identification of prokaryotic and eukaryotic signal peptides and prediction of their cleavage sites Protein Eng 1997 10 1 1 6 10.1093/protein/10.1.1 9051728

[B23] Emanuelsson O Nielsen H Brunak S von Heijne G Predicting subcellular localization of proteins based on their N-terminal amino acid sequence J Mol Biol 2000 300 4 1005 1016 10.1006/jmbi.2000.3903 10891285

[B24] Nakai K Horton P PSORT: a program for detecting sorting signals in proteins and predicting their subcellular localization Trends Biochem Sci 1999 24 1 34 36 10.1016/S0968-0004(98)01336-X 10087920

[B25] Livak KJ Schmittgen TD Analysis of relative gene expression data using real-time quantitative PCR and the 2(-Delta Delta C(T)) Method Methods 2001 25 4 402 408 10.1006/meth.2001.1262 11846609

[B26] Bar-Or C Czosnek H Koltai H Cross-species microarray hybridizations: a developing tool for studying species diversity Trends Genet 2007 23 4 200 207 10.1016/j.tig.2007.02.003 17313995

[B27] Buckley BA Comparative environmental genomics in non-model species: using heterologous hybridization to DNA-based microarrays J Exp Biol 2007 210 Pt 9 1602 1606 10.1242/jeb.002402 17449825

[B28] Kang JS Kim JS Zinc finger proteins as designer transcription factors J Biol Chem 2000 275 12 8742 8748 10.1074/jbc.275.12.8742 10722717

[B29] Taylor AW Review of the activation of TGF-beta in immunity J Leukoc Biol 2009 85 1 29 33 10.1189/jlb.0708415 18818372 PMC3188956

[B30] Mangan MS Kaiserman D Bird PI The role of serpins in vertebrate immunity Tissue Antigens 2008 72 1 1 10 10.1111/j.1399-0039.2008.01059.x 18498291

[B31] Kang CB Hong Y Dhe-Paganon S Yoon HS FKBP family proteins: immunophilins with versatile biological functions Neurosignals 2008 16 4 318 325 10.1159/000123041 18635947

[B32] Ma D Carlow CK Molecular characterization of FKBP13 from filarial parasites Mol Biochem Parasitol 1999 99 2 263 267 10.1016/S0166-6851(99)00013-4 10340490

[B33] Wan YY Flavell RA TGF-beta and regulatory T cell in immunity and autoimmunity J Clin Immunol 2008 28 6 647 659 10.1007/s10875-008-9251-y 18792765 PMC2837280

[B34] Gomez-Escobar N Gregory WF Maizels RM Identification of tgh-2, a filarial nematode homolog of *Caenorhabditis elegans *daf-7 and human transforming growth factor beta, expressed in microfilarial and adult stages of *Brugia malayi* Infect Immun 2000 68 11 6402 6410 10.1128/IAI.68.11.6402-6410.2000 11035752 PMC97726

[B35] Kim DS Park SH Park KC Transforming growth factor-beta1 decreases melanin synthesis via delayed extracellular signal-regulated kinase activation Int J Biochem Cell Biol 2004 36 8 1482 1491 15147727 10.1016/j.biocel.2003.10.023

[B36] Martinez-Esparza M Jimenez-Cervantes C Beermann F Aparicio P Lozano JA Garcia-Borron JC Transforming growth factor-beta1 inhibits basal melanogenesis in B16/F10 mouse melanoma cells by increasing the rate of degradation of tyrosinase and tyrosinase-related protein-1 J Biol Chem 1997 272 7 3967 3972 10.1074/jbc.272.7.3967 9020101

[B37] van Gent D Sharp P Morgan K Kalsheker N Serpins: structure, function and molecular evolution Int J Biochem Cell Biol 2003 35 11 1536 1547 10.1016/S1357-2725(03)00134-1 12824063

[B38] Cerenius L Soderhall K The prophenoloxidase-activating system in invertebrates Immunol Rev 2004 198 116 126 10.1111/j.0105-2896.2004.00116.x 15199959

[B39] Nappi AJ Frey F Carton Y Drosophila serpin 27A is a likely target for immune suppression of the blood cell-mediated melanotic encapsulation response J Insect Physiol 2005 51 2 197 205 10.1016/j.jinsphys.2004.10.013 15749104

[B40] Stanley P Stein PE BmSPN2, a serpin secreted by the filarial nematode *Brugia malayi*, does not inhibit human neutrophil proteinases but plays a noninhibitory role Biochemistry 2003 42 20 6241 6248 10.1021/bi0271650 12755628

[B41] Zang X Yazdanbakhsh M Jiang H Kanost MR Maizels RM A novel serpin expressed by blood-borne microfilariae of the parasitic nematode *Brugia malayi *inhibits human neutrophil serine proteinases Blood 1999 94 4 1418 1428 10438730

[B42] Michel K Budd A Pinto S Gibson TJ Kafatos FC *Anopheles gambiae *SRPN2 facilitates midgut invasion by the malaria parasite *Plasmodium berghei* EMBO Rep 2005 6 9 891 897 10.1038/sj.embor.7400478 16113656 PMC1369158

[B43] Michel K Suwanchaichinda C Morlais I Lambrechts L Cohuet A Awono-Ambene PH Simard F Fontenille D Kanost MR Kafatos FC Increased melanizing activity in *Anopheles gambiae *does not affect development of *Plasmodium falciparum* Proc Natl Acad Sci USA 2006 103 45 16858 16863 10.1073/pnas.0608033103 17065316 PMC1636544

[B44] Ghedin E Wang S Spiro D Caler E Zhao Q Crabtree J Allen JE Delcher AL Guiliano DB Miranda-Saavedra D Angiuoli SV Creasy T Amedeo P Haas B El-Sayed NM Wortman JR Feldblyum T Tallon L Schatz M Shumway M Koo H Salzberg SL Schobel S Pertea M Pop M White O Barton GJ Carlow CK Crawford MJ Daub J Dimmic MW Estes CF Foster JM Ganatra M Gregory WF Johnson NM Jin J Komuniecki R Korf I Kumar S Laney S Li BW Li W Lindblom TH Lustigman S Ma D Maina CV Martin DM McCarter JP McReynolds L Mitreva M Nutman TB Parkinson J Peregrín-Alvarez JM Poole C Ren Q Saunders L Sluder AE Smith K Stanke M Unnasch TR Ware J Wei AD Weil G Williams DJ Zhang Y Williams SA Fraser-Liggett C Slatko B Blaxter ML Scott AL Draft genome of the filarial nematode parasite *Brugia malayi* Science 2007 317 5845 1756 1760 10.1126/science.1145406 17885136 PMC2613796

[B45] Wolfinger RD Gibson G Wolfinger ED Bennett L Hamadeh H Bushel P Afshari C Paules RS Assessing gene significance from cDNA microarray expression data via mixed models J Comput Biol 2001 8 6 625 637 10.1089/106652701753307520 11747616

[B46] Kerr MK Linear models for microarray data analysis: hidden similarities and differences J Comput Biol 2003 10 6 891 901 10.1089/106652703322756131 14980016

[B47] Benjamini Y Drai D Elmer G Kafkafi N Golani I Controlling the false discovery rate in behavior genetics research Behav Brain Res 2001 125 1-2 279 284 10.1016/S0166-4328(01)00297-2 11682119

[B48] Storey JD Tibshirani R Statistical significance for genomewide studies Proc Natl Acad Sci USA 2003 100 16 9440 9445 10.1073/pnas.1530509100 12883005 PMC170937

[B49] Kanehisa M Goto S KEGG: kyoto encyclopedia of genes and genomes Nucleic Acids Res 2000 28 1 27 30 10.1093/nar/28.1.27 10592173 PMC102409

[B50] Emanuelsson O Brunak S von Heijne G Nielsen H Locating proteins in the cell using TargetP, SignalP and related tools Nat Protoc 2007 2 4 953 971 10.1038/nprot.2007.131 17446895

