# Supplementary material for: Differential transcript expression between the microfilariae of the filarial nematodes, Brugia malayi and B. pahangi
Source: BMC Genomics. 2010 Apr 7;11:225. doi: 10.1186/1471-2164-11-225 (PMC2874553; doi:10.1186/1471-2164-11-225)
Supplement: Additional file 1 — Brugia gene transcripts that are up-regulated in B. pahangi microfilariae. This file contains a list of genes represented by probes on the microarray that had signal intensity ratios of 2 or higher in B. pahangi microfilariae and a FDR estimate of 5%. These genes were considered preferentially expressed and manually mapped into the appropriate KEGG pathway. a Genes represented by more than one probe on the BmV2 array. For these genes, an average of the p-value and signal intensity ratio is shown. Genes in each of the KEGG pathway groups are ranked according to their ratios. [file 1471-2164-11-225-S1.DOC]

| **Pub Locus** | **Annotation and KEGG Pathway** | ***p*-value** | **Bp/Bm** |
| --- | --- | --- | --- |
|  |  |  |  |
| **Cellular Processes (14 genes)** | |  |  |
| Bm1_03995^a^ | Serpin (BmSERPIN), putative | 4.75E-06 | 5.05 |
| Bm1_02070^a^ | Serpin, putative | 1.28E-04 | 3.85 |
| Bm1_43925 | Beta-NAC-like protein, putative | 1.30E-05 | 3.61 |
| Bm1_03865^a^ | Dynein light intermediate chain, putative | 1.96E-04 | 2.82 |
| Bm1_30645^a^ | Pregnancy-associated plasma protein-A, putative | 6.99E-06 | 2.70 |
| Bm1_18845 | GRIM-19 protein | 2.00E-04 | 2.34 |
| Bm1_47770 | p25-alpha family protein | 2.43E-06 | 2.34 |
| Bm1_37335 | Ankyrin-related unc-44, putative | 2.33E-04 | 2.29 |
| Bm1_37310^a^ | FKBP-type peptidyl-prolyl cis-trans isomerase-13, BmFKBP-13 | 3.72E-05 | 2.28 |
| Bm1_49010 | FKBP-type peptidyl-prolyl cis-trans isomerase-12, BmFKBP-12 | 5.09E-04 | 2.25 |
| Bm1_19920 | Kinesin light chain (KLC), putative | 4.96E-06 | 2.25 |
| Bm1_25935 | Serpin, putative | 9.48E-05 | 2.19 |
| Bm1_11180 | Uncoordinated protein 1, putative | 2.19E-06 | 2.15 |
| Bm1_56195 | Transforming growth factor b homolog, putative | 1.09E-04 | 2.02 |
| **Environmental Information Processing (9 genes)** | |  |  |
| Bm1_42715^a^ | GTP-binding protein, identical | 2.58E-06 | 6.31 |
| Bm1_49190 | Ras family protein | 3.27E-08 | 3.42 |
| Bm1_31500 | Phosphatidylethanolamine-binding protein | 2.71E-06 | 3.11 |
| Bm1_24145 | ABC1 family protein | 8.18E-05 | 3.03 |
| Bm1_46135 | AF2 peptide, putative | 4.83E-06 | 2.64 |
| Bm1_35155 | Integrin cytoplasmic domain-associated protein 1, isoform 1, putative | 7.15E-05 | 2.46 |
| Bm1_39915 | Transthyretin-like family protein | 1.60E-04 | 2.22 |
| Bm1_37165 | RhoGAP domain containing protein | 2.51E-03 | 2.03 |
| Bm1_45355 | Calreticulin family protein | 2.43E-05 | 2.01 |
| **Genetic Information Processing (37 genes)** | |  |  |
| Bm1_49585 | 60S ribosomal protein L31, putative | 2.60E-08 | 7.85 |
| Bm1_40080 | 40S ribosomal protein S2, putative | 3.46E-08 | 5.71 |
| Bm1_41485 | Probable Sin3 associated polypeptide p18, putative | 5.52E-06 | 3.22 |
| Bm1_57630 | Retinoblastoma-binding protein putative | 1.78E-06 | 3.20 |
| Bm1_35975 | 60S ribosome subunit biogenesis protein NIP7, putative | 6.96E-07 | 3.18 |
| Bm1_53665 | Translin family protein | 1.46E-06 | 3.14 |
| Bm1_02405^a^ | Fidgetin protein, putative | 8.44E-06 | 3.12 |
| Bm1_13955 | Trafficking protein particle complex subunit 1, putative | 2.17E-04 | 3.09 |
| Bm1_50275 | U6 snRNA-associated Sm-like protein LSm5, putative | 2.68E-05 | 3.08 |
| Bm1_53990 | Zinc finger, C2H2 type family protein | 9.69E-07 | 2.97 |
| Bm1_02420 | ATPase, AAA family protein | 5.92E-07 | 2.93 |
| Bm1_20430 | Cleavage and polyadenylation specificity factor, putative | 6.60E-07 | 2.87 |
| Bm1_48910 | eIF2 gamma, putative | 1.16E-06 | 2.87 |
| Bm1_02505 | Histone H2A, putative | 2.80E-04 | 2.84 |
| Bm1_00195 | 40S ribosomal protein S26, putative | 4.08E-07 | 2.68 |
| Bm1_49275 | Mitochondrial 28S ribosomal protein S33, putative | 2.77E-04 | 2.68 |
| Bm1_48955^a^ | COMM domain containing 4, putative | 1.58E-05 | 2.67 |
| Bm1_28420 | DnaJ protein, putative | 7.45E-06 | 2.62 |
| Bm1_34005 | RNA polymerase II, putative | 8.90E-04 | 2.48 |
| Bm1_00185^a^ | 40S ribosomal protein S26, putative | 2.76E-04 | 2.46 |
| Bm1_43700^a^ | Putative LAG1-interacting protein | 9.11E-06 | 2.44 |
| Bm1_44745 | 26S proteasome non-ATPase regulatory subunit Nin1/mts3 family protein | 1.72E-06 | 2.40 |
| Bm1_50605 | Ribonuclease T2 family protein | 1.78E-06 | 2.40 |
| Bm1_28015^a^ | Coatomer epsilon subunit family protein | 1.77E-04 | 2.39 |
| Bm1_48170 | NTF2-related export protein, putative | 9.68E-05 | 2.39 |
| Bm1_27610 | RNA binding protein, putative | 4.72E-06 | 2.34 |
| Bm1_19765 | Helix-loop-helix DNA-binding domain containing protein | 1.66E-06 | 2.31 |
| Bm1_09350 | Threonyl-tRNA synthetase, cytoplasmic, putative | 5.00E-04 | 2.28 |
| Bm1_04800 | 60S ribosomal protein L23a, putative | 5.48E-04 | 2.27 |
| Bm1_02495 | Histone H3, putative | 2.15E-05 | 2.25 |
| Bm1_30975 | Zinc finger C-x8-C-x5-C-x3-H type containing protein, putative | 1.69E-05 | 2.17 |
| Bm1_19805 | Small heat shock protein, putative | 7.24E-04 | 2.14 |
| Bm1_18875 | TB2/DP1, HVA22 family protein | 1.39E-04 | 2.10 |
| Bm1_51245 | DEAD/DEAH box helicase family protein | 2.37E-05 | 2.06 |
| Bm1_44270 | GATA zinc finger family protein | 2.82E-07 | 2.05 |
| Bm1_25015 | Hematopoietic stem/progenitor cells 176, putative | 2.83E-04 | 2.05 |
| Bm1_49245 | Sec8 exocyst complex component specific domain containing protein | 1.27E-04 | 2.04 |
| **Metabolism (16 genes)** | |  |  |
| Bm1_55690 | NADH-ubiquinone oxidoreductase B12 subunit , putative | 3.02E-06 | 3.26 |
| Bm1_48330 | Dihydrofolate reductase, putative | 1.59E-05 | 3.07 |
| Bm1_44235 | GMP synthase, putative | 1.01E-04 | 2.88 |
| Bm1_30745 | Dual specificity phosphatase, catalytic domain containing protein | 5.45E-06 | 2.75 |
| Bm1_25860 | Probable pyruvate dehydrogenase E1 component alpha subunit, mitochondrial, putative | 6.96E-05 | 2.66 |
| Bm1_45510 | MFP3, putative | 2.68E-06 | 2.56 |
| Bm1_33495 | Probable protein disulfide isomerase A6 precursor, putative | 1.90E-05 | 2.46 |
| Bm1_36665 | Fumarylacetoacetate hydrolase domain containing 1, putative | 1.55E-06 | 2.33 |
| Bm1_44205 | V-type ATPase 116kDa subunit family protein | 1.90E-03 | 2.31 |
| Bm1_32635 | dTDP-4-dehydrorhamnose 3,5-epimerase, putative | 2.60E-05 | 2.24 |
| Bm1_47735^a^ | Lipase family protein | 8.66E-05 | 2.22 |
| Bm1_43195 | ADP-specific Phosphofructokinase/Glucokinase conserved region family protein | 1.61E-05 | 2.18 |
| Bm1_43565 | MBOAT family protein | 5.67E-06 | 2.10 |
| Bm1_32890 | Histidine acid phosphatase family protein | 1.60E-05 | 2.08 |
| Bm1_35885 | MFP2, putative | 1.12E-06 | 2.08 |
| Bm1_32535^a^ | Putative carbonic anhydrase 5 precursor, putative | 1.15E-04 | 2.05 |
| **Unknown Pathway (47 genes)** | |  |  |
| Bm1_11050^a^ | Hypothetical protein | 8.33E-08 | 7.37 |
| Bm1_04670^a^ | Serine-rich protein-like | 4.01E-08 | 6.41 |
| Bm1_09135^a^ | Hypothetical protein | 2.91E+00 | 5.84 |
| Bm1_46900 | Hypothetical protein | 3.90E-07 | 4.44 |
| Bm1_55110 | Hypothetical protein | 4.16E-06 | 3.84 |
| Bm1_35775 | Hypothetical 44.2 kDa protein in RME1-TFC4 intergenic region, putative | 1.04E-06 | 3.74 |
| Bm1_30675 | DREV methyltransferase family protein | 7.60E-06 | 3.02 |
| Bm1_49220 | Hypothetical UPF0027 protein F16A11.2 in chromosome I, putative | 2.97E-05 | 3.01 |
| Bm1_49635 | Protein C17H12.11, putative | 7.14E-07 | 3.00 |
| Bm1_49810 | Conserved hypothetical protein | 1.07E-06 | 2.93 |
| Bm1_51005 | Hypothetical protein | 4.18E-07 | 2.92 |
| Bm1_36985 | Conserved hypothetical protein, putative | 5.70E-08 | 2.91 |
| Bm1_35905 | Hypothetical protein | 4.55E-06 | 2.91 |
| Bm1_41020^a^ | LD41395p, putative | 8.31E-06 | 2.77 |
| Bm1_46755 | AEL166Cp, putative | 5.32E-06 | 2.73 |
| Bm1_44285 | Hypothetical protein | 1.37E-06 | 2.73 |
| Bm1_44755 | RE21922p, putative | 2.57E-06 | 2.71 |
| Bm1_30680 | Hypothetical protein | 7.60E-06 | 2.70 |
| Bm1_11605 | Conserved hypothetical protein | 1.42E-05 | 2.69 |
| Bm1_31215^a^ | Conserved hypothetical protein | 6.16E-06 | 2.63 |
| Bm1_48510^a^ | Hypothetical protein | 3.56E-04 | 2.61 |
| Bm1_42620 | DNA segment, Chr 7, Wayne State University 180, expressed, putative | 1.01E-04 | 2.57 |
| Bm1_50320 | Hypothetical protein | 4.07E-05 | 2.54 |
| Bm1_54485 | CHCH domain containing protein | 3.48E-05 | 2.47 |
| Bm1_07905 | Metallophosphoesterase 1, putative | 3.85E-05 | 2.39 |
| Bm1_29060 | RIKEN cDNA A930016P21, putative | 1.84E-04 | 2.34 |
| Bm1_42865 | Brugia malayi antigen, putative | 1.72E-06 | 2.33 |
| Bm1_23010 | Hypothetical protein | 1.60E-05 | 2.31 |
| Bm1_07705 | Hypothetical protein | 1.95E-05 | 2.26 |
| Bm1_41610 | Within the bgcn gene intron protein, putative | 7.03E-05 | 2.26 |
| Bm1_24190 | Hypothetical protein | 6.96E-05 | 2.20 |
| Bm1_45770^a^ | Hypothetical 31.4 kDa protein T19C3.2 in chromosome III, putative | 2.40E-06 | 2.19 |
| Bm1_00735^a^ | Hypothetical protein | 1.59E-04 | 2.19 |
| Bm1_52330 | Ser/Thr-rich protein T10 in DGCR region, putative | 7.49E-07 | 2.19 |
| Bm1_42730 | Conserved hypothetical protein | 4.43E-05 | 2.18 |
| Bm1_02045 | Cysteine-rich protein 1, putative | 1.85E-04 | 2.16 |
| Bm1_29610 | Hypothetical protein | 6.56E-07 | 2.16 |
| Bm1_23875^a^ | Hypothetical protein | 1.21E-05 | 2.14 |
| Bm1_11270 | Hypothetical protein | 3.34E-04 | 2.13 |
| Bm1_47675 | Hypothetical protein | 1.86E-06 | 2.09 |
| Bm1_40635 | Conserved hypothetical protein | 1.96E-04 | 2.07 |
| Bm1_34000 | Hypothetical protein | 1.64E-03 | 2.05 |
| Bm1_53715 | TM2 domain containing protein | 4.31E-06 | 2.04 |
| Bm1_20145 | Hypothetical protein | 3.05E-05 | 2.03 |
| Bm1_40630 | Hypothetical protein | 2.48E-05 | 2.02 |
| Bm1_50160 | Hypothetical protein | 2.40E-04 | 2.02 |
| Bm1_21680 | Hypothetical protein | 5.18E-06 | 2.01 |
