# Supplementary material for: Differential transcript expression between the microfilariae of the filarial nematodes, Brugia malayi and B. pahangi
Source: BMC Genomics. 2010 Apr 7;11:225. doi: 10.1186/1471-2164-11-225 (PMC2874553; doi:10.1186/1471-2164-11-225)
Supplement: Additional file 2 — Brugia gene transcripts that are up-regulated in B. malayi microfilariae. This file contains a list of genes represented by probes on the microarray that had signal intensity ratios of 2 or higher in B. malayi microfilariae and a FDR estimate of 5%. These genes were considered preferentially expressed and manually mapped into the appropriate KEGG pathway. a Genes represented by more than one probe on the BmV2 array. For these genes, an average of the p-value and signal intensity ratio is shown. Genes in each of the KEGG pathway groups are ranked according to their ratios. [file 1471-2164-11-225-S2.DOC]

| **Pub Locus** | **Annotation and KEGG Pathway** | ***p*-value** | **Bm/Bp** |
| --- | --- | --- | --- |
|  | |  |  |
| **Cellular Processes (9 genes)** | |  |  |
| Bm1_44655 | Fukutin, putative | 1.20E-08 | 9.13 |
| Bm1_49400^a^ | Myosin VI, putative | 1.81E-05 | 6.07 |
| Bm1_57380 | Pescadillo N-terminus family protein | 1.06E-04 | 3.08 |
| Bm1_56360^a^ | Nematode cuticle collagen N-terminal domain containing protein | 7.73E-06 | 2.74 |
| Bm1_08135 | Cell division protein kinase 10, putative | 9.48E-06 | 2.57 |
| Bm1_04450^a^ | Paramyosin, putative | 1.31E-05 | 2.57 |
| Bm1_43315 | Troponin C, isoform 2, putative | 9.86E-06 | 2.14 |
| Bm1_06730 | Snowski, identical | 9.85E-05 | 2.13 |
| Bm1_55930 | Nematode cuticle collagen N-terminal domain containing protein | 6.02E-04 | 2.05 |
| **Environmental Information Processing (6 genes)** | |  |  |
| Bm1_50670 | Potassium channel chain n2P18 homolog, putative | 4.27E-08 | 9.95 |
| Bm1_40190 | DXImx40e protein, putative | 4.22E-04 | 2.13 |
| Bm1_35705 | Ras association | 6.03E-05 | 2.11 |
| Bm1_32085 | Rab6, putative | 9.56E-05 | 2.09 |
| Bm1_12150 | Chloride intracellular channel exc-4 (Excretory canal abnormal protein4), putative | 2.01E-03 | 2.06 |
| Bm1_12070 | K+ channel tetramerisation domain containing protein | 8.44E-04 | 2.00 |
| **Genetic Information Processing (38 genes)** | |  |  |
| Bm1_22980 | Zinc finger, C2H2 type family protein | 6.73E-07 | 6.58 |
| Bm1_36235^a^ | Asparaginyl-tRNA synthetase, cytoplasmic, putative | 2.49E-05 | 6.36 |
| Bm1_04360 | Zinc finger, C2H2 type family protein | 6.11E-07 | 5.66 |
| Bm1_21445^a^ | Zinc finger, C2H2 type family protein | 1.36E-07 | 5.64 |
| Bm1_47425 | Zinc finger, C2H2 type family protein | 7.54E-07 | 5.31 |
| Bm1_01315^a^ | Zinc finger, C2H2 type family protein | 7.81E-06 | 5.11 |
| Bm1_35265 | Zinc finger, C2H2 type family protein | 4.99E-07 | 4.91 |
| Bm1_23300^a^ | Zinc finger, C2H2 type family protein | 3.41E-07 | 4.89 |
| Bm1_09090^a^ | Zinc finger, C2H2 type family protein | 4.85E-05 | 4.84 |
| Bm1_55700^a^ | KH domain containing protein | 5.43E-07 | 4.72 |
| Bm1_13725 | Zinc finger, C2H2 type family protein | 1.49E-06 | 4.38 |
| Bm1_00290^a^ | Zinc finger, C2H2 type family protein | 4.11E-05 | 3.69 |
| Bm1_08820 | Zinc finger protein 207, putative | 1.14E-07 | 3.64 |
| Bm1_07320 | Zinc finger, C2H2 type family protein | 1.59E-05 | 3.61 |
| Bm1_04225 | Zinc finger, C2H2 type family protein | 7.47E-06 | 3.42 |
| Bm1_11185 | Zinc finger, C2H2 type family protein | 4.56E-06 | 3.34 |
| Bm1_03710 | Zinc finger, C2H2 type family protein | 2.99E-06 | 3.32 |
| Bm1_15340 | Zinc finger, C2H2 type family protein | 2.71E-06 | 3.23 |
| Bm1_39920 | Homologous to Bombyx mori multiprotein bridging factor, putative | 5.21E-07 | 3.22 |
| Bm1_29545^a^ | Zinc finger, C2H2 type family protein | 1.02E-04 | 3.00 |
| Bm1_18365 | Zinc finger, C2H2 type family protein | 2.55E-06 | 2.75 |
| Bm1_02255 | Zinc finger, C2H2 type family protein | 1.99E-05 | 2.72 |
| Bm1_10870^a^ | Zinc finger, C2H2 type family protein | 1.03E-04 | 2.67 |
| Bm1_32770 | Initiation factor 2 subunit family protein | 3.90E-07 | 2.65 |
| Bm1_12455^a^ | Zinc finger, C2H2 type family protein | 1.86E-04 | 2.64 |
| Bm1_13985 | Zinc finger, C2H2 type family protein | 9.63E-05 | 2.64 |
| Bm1_04220^a^ | Zinc finger, C2H2 type family protein | 3.89E-04 | 2.63 |
| Bm1_54245 | Hypoxia-induced factor 1, putative | 2.35E-04 | 2.53 |
| Bm1_05155 | Zinc finger, C2H2 type family protein | 1.08E-03 | 2.53 |
| Bm1_48535 | Microsomal signal peptidase 21 kDa subunit, putative | 2.16E-06 | 2.52 |
| Bm1_08145 | Zinc finger, C2H2 type family protein | 2.31E-03 | 2.49 |
| Bm1_31055 | RNA recognition motif containing protein, putative | 5.37E-04 | 2.31 |
| Bm1_43180 | RNA polymerase Rpb1, domain 2 family protein | 1.06E-04 | 2.25 |
| Bm1_41735 | 60S ribosomal protein L24, putative | 1.28E-04 | 2.24 |
| Bm1_03295 | Brix domain containing protein 1 homolog, putative | 1.84E-04 | 2.14 |
| Bm1_26995 | DNA-directed RNA polymerases I, II, and III 17.1 kDa polypeptide, putative | 6.55E-04 | 2.14 |
| Bm1_49040 | DEAD, putative | 1.41E-05 | 2.05 |
| Bm1_31435 | SNF2 family N-terminal domain containing protein | 1.75E-06 | 2.05 |
| **Metabolism (8 genes)** | |  |  |
| Bm1_01300^a^ | Alpha amylase, catalytic domain containing protein | 1.78E-07 | 6.73 |
| Bm1_22105^a^ | Acyl-CoA desaturase, putative | 8.14E-07 | 3.99 |
| Bm1_50530^a^ | Aminopeptidase W07G4.4 in chromosome V, putative | 4.57E-05 | 2.99 |
| Bm1_35135 | Amino acid permease family protein | 2.13E-04 | 2.45 |
| Bm1_24115 | Enolase, putative | 3.73E-06 | 2.18 |
| Bm1_01585^a^ | Fatty acid elongation protein 3, putative | 4.03E-04 | 2.14 |
| Bm1_40960 | Chymotrypsin/elastase isoinhibitors 2 to 5, putative | 2.21E-04 | 2.09 |
| Bm1_56305^a^ | Leucyl aminopeptidase, putative | 2.46E-05 | 2.09 |
| **Unknown Pathway (46 genes)** | |  |  |
| Bm1_06065^a^ | Hypothetical protein | 1.21E-06 | 7.26 |
| Bm1_44495 | Hypothetical protein | 3.17E-08 | 6.69 |
| Bm1_04615 | Hypothetical protein | 4.79E-04 | 6.20 |
| Bm1_38635 | Conserved hypothetical protein | 3.42E-08 | 5.67 |
| Bm1_22110 | Hypothetical protein | 1.04E-07 | 4.70 |
| Bm1_40360 | Conserved hypothetical protein | 3.00E-08 | 4.05 |
| Bm1_14020^a^ | Hypothetical protein | 4.01E-04 | 4.01 |
| Bm1_22600 | Hypothetical protein | 8.76E-06 | 3.51 |
| Bm1_02835 | Conserved hypothetical protein | 7.83E-06 | 3.28 |
| Bm1_32660 | Hypothetical protein | 2.58E-06 | 3.16 |
| Bm1_25950 | Hypothetical protein | 1.31E-06 | 3.13 |
| Bm1_14030 | Hypothetical protein | 1.20E-05 | 3.01 |
| Bm1_17145 | Hypothetical protein | 7.40E-05 | 2.85 |
| Bm1_06490 | Hypothetical protein | 7.08E-08 | 2.80 |
| Bm1_09500 | Hypothetical protein | 1.00E-05 | 2.80 |
| Bm1_43860 | Hypothetical protein | 2.76E-05 | 2.78 |
| Bm1_07515 | Hypothetical protein | 6.64E-07 | 2.77 |
| Bm1_34385 | Hypothetical protein | 1.84E-04 | 2.70 |
| Bm1_30525^a^ | Hypothetical protein | 1.04E-05 | 2.64 |
| Bm1_03355 | Hypothetical protein | 1.40E-04 | 2.63 |
| Bm1_48565 | Gut on exterior protein 2, putative | 1.61E-06 | 2.59 |
| Bm1_33830 | Prismalin-14, putative | 4.08E-04 | 2.55 |
| Bm1_31930^a^ | Hypothetical protein | 4.11E-04 | 2.53 |
| Bm1_04830 | 15 kDa selenoprotein precursor, putative | 1.86E-06 | 2.51 |
| Bm1_21005 | Hypothetical protein | 4.08E-04 | 2.46 |
| Bm1_22820 | Hypothetical protein | 4.81E-05 | 2.44 |
| Bm1_43580 | Hypothetical protein | 7.36E-04 | 2.44 |
| Bm1_44220^a^ | Conserved hypothetical protein | 4.63E-04 | 2.42 |
| Bm1_33955 | Hypothetical protein | 5.46E-05 | 2.37 |
| Bm1_40040 | Hypothetical protein | 1.52E-05 | 2.36 |
| Bm1_05760 | Hypothetical protein | 4.15E-05 | 2.35 |
| Bm1_03570 | Hypothetical protein | 1.01E-03 | 2.34 |
| Bm1_22360 | DB module family protein | 1.16E-03 | 2.30 |
| Bm1_19100 | Major microfilarial sheath protein precursor, putative | 7.15E-06 | 2.28 |
| Bm1_47450 | Conserved hypothetical protein, putative | 8.55E-04 | 2.26 |
| Bm1_45345 | Hypothetical 31.4 kDa protein T19C3.2 in chromosome III, putative | 6.14E-07 | 2.26 |
| Bm1_00295 | Hypothetical protein | 1.16E-04 | 2.25 |
| Bm1_03735^a^ | Hypothetical protein | 6.28E-04 | 2.23 |
| Bm1_37655 | Hypothetical protein | 2.03E-04 | 2.20 |
| Bm1_22295 | Conserved hypothetical protein | 2.54E-03 | 2.19 |
| Bm1_49615 | Hypothetical protein | 4.66E-04 | 2.18 |
| Bm1_25390 | Hypothetical protein | 6.68E-06 | 2.16 |
| Bm1_39435 | Hypothetical protein | 2.19E-04 | 2.15 |
| Bm1_46180 | Hypothetical protein | 5.96E-06 | 2.11 |
| Bm1_43650 | Hypothetical protein | 6.48E-04 | 2.07 |
| Bm1_24130 | Hypothetical protein | 2.01E-03 | 2.05 |
